# Smoking increases the risk of infectious diseases: A narrative review

**DOI:** 10.18332/tid/123845

**Published:** 2020-07-14

**Authors:** Chen Jiang, Qiong Chen, Mingxuan Xie

**Affiliations:** 1 Department of Gerontology and Respirology, Xiangya Hospital, Central South University, Changsha, China; 2 National Clinical Research Centre for Geriatric Disorders, Department of Geriatrics, Xiangya Hospital, Central South University, Changsha, China

**Keywords:** smoking, tobacco, infection, respiratory tract

## Abstract

Smoking is relevant to infectious diseases resulting in increased prevalence and mortality. In this article, we aim to provide an overview of the effects of smoking in various infections and to explain the potential mechanisms. We searched PubMed and other relevant databases for scientific studies that explored the relationship between smoking and infection. The mechanisms of susceptibility to infection in smokers may include alteration of the structural, functional and immunologic host defences. Smoking is one of the main risk factors for infections in the respiratory tract, digestive tract, reproductive tract, and other systems in humans, increasing the prevalence of HIV, tuberculosis, SARS-CoV, and the current SARS-CoV-2. Smoking cessation can reduce the risk of infection. Smoking increases the incidence of infections and aggravates the progress and prognosis of infectious diseases in a dose-dependent manner. Smoking cessation promotion and education are the most practical and economical preventive measures to reduce aggravation of disease infection owing to tobacco use.

## INTRODUCTION

Smoking is one of the most severe public health problems in the world. According to the WHO Global report on trends in prevalence of tobacco use 2000– 2025, smoking accounts for 9% of all deaths worldwide, and more than half of smokers die from smoking-related diseases^1^. China is the largest producer and consumer of tobacco in the world. In 2018, about 26.6% of adults (aged ≥15 years; 308 million in total) were current smokers and 44.9% of adults (515 million) were exposed to secondhand smoke at home^1^. Exposure to cigarette smoke and secondhand smoke is very harmful to human health. There are over 7000 chemical components in tobacco smoke, of these, over 250 are toxic or carcinogenic, such as aldehydes, nitrides, and others that irritate the respiratory tract. Nicotine leads to tobacco addiction; benzpyrene, arsenic, cadmium and other components have carcinogenic effects and nitric oxide can reduce oxygen transport by erythrocytes. Smoking can damage nearly all organs of the human body and is one of the main risk factors for respiratory infection and infectious diseases in other systems, in a dose-dependent manner^2,3^. In this review, we discuss the mechanisms and influence of smoking on infections in various systems, including the respiratory system, digestive system, and genital system, among others, in order to provide evidence for the active promotion of smoking cessation.

## DEVELOPMENTS

### Mechanisms of susceptibility

The potential mechanisms of how cigarette smoke increases the risk of systemic infections are not completely understood. These may include alteration of the structural, functional and immunologic host defences^2^.

#### Structural and functional changes

Harmful substances in cigarette smoke can interfere with the structure and function of the respiratory tract, the oral environment and the digestive tract, facilitating invasion by pathogenic organisms and increasing susceptibility to infections.

##### Smoking and the respiratory tract

The respiratory epithelium constitutes the first line of defence against inhaled pollutants and pathogens. Cigarette smoke can directly damage the airway epithelial barrier, including ciliated cells, goblet cells, basal cells, and submucosal secretory glands^4^. Toxic substances in cigarette smoke, as well as nicotine containing electronic cigarettes (e-cigarettes), can impair the continuity of ciliary oscillation^5^, and lead to mucus hypersecretion^6^, delayed mucociliary clearance^7^, which is conducive to the colonization by and the reproduction of pathogens^8^. Relevant to these abnormalities, cigarette smoke perturbs the metabolism of human airway basal stem/progenitor cells, and affects the replenishment of mucociliary eplithelium^9^. Cigarette smoke can also impair the integrity of the airway epithelium, mainly by disruption of intercellular contacts^10^. The use of cigarettes^11^ and e-cigarettes^12^ diminishes cough reflex sensitivity in humans^13^, preventing the elimination of pathogens. Fortunately, cough sensitivity can be gradually restored as early as 2 weeks after smoking cessation^14^.

The above structure alteration and dysfunction may be explained by the following reasons. Genetic changes, such as loss of heterozygosity (LOH) and microsatellite instability (MSI), can be found in the bronchial epithelium of current or former smokers^15^. Cigarette smoking causes persistent DNA damage and inefficient DNA repair of epithelial lung cells, resulting in somatic mutations, as a form of LOH and MSI^16-18^. The accumulation of mutation induces cell death and tissue destruction, leading to barrier impairment and structural changes like metaplasia, dysplasia and airway structural reform^19,20^. Persistent or inefficient autophagy may be detrimental to lung epithelial cells, promoting lung injury^21^. Exposure to cigarette smoke is confirmed as a main contributor to the induction of mitochondrial dysfunction and mitophagy impairment, along with the accumulation of damaged mitochondrial DNA^22^. Enhanced mitophagy can cause injury and senescence in airway epithelial cells, impairing the barrier integrity. Insufficient mitophagy may exaggerate airway wall thickening and emphysematous changes, contributing to structural changes and increased susceptibility to infection^23-25^. The reactive oxygen species (ROS) in cigarette smoke disturbs mitochondrial function in airway epithelial cells by decreasing the ability of mitochondria for ATP synthesis. Mitochondrial disfunction leads to cellular necrosis and progressive inflammation in the lungs, promoting tissue remodeling and susceptibility to infection^26-29^. Nitric oxide is endogenously released in the airways by nitric oxide synthase, and can interact with ROS and oxygen free radicals to form other reactive nitrogen species (RNS) after inhalation of cigarette smoke, all of which are essential in the killing of invading microorganisms. In addition, smoking can decrease the activity of endothelial nitric oxide synthase^30^, leading to decreased lung protection function. Enhanced levels of ROS and RNS by cigarette smoking are able to compromise cell function by damaging DNA, lipids, proteins, and carbohydrates, inducing several pathophysiological conditions such as apoptosis and necrosis^31,32^. The large amounts of free radicals in cigarette smoke can damage the integrity of respiratory tract and alveolar epithelial cells, leading to an increased likelihood of infection.

##### Oral environment

Smoking is associated with excessive destruction of the supporting periodontal tissues, resulting in bone loss, pocket formation, and premature tooth loss^33^. Cigarette smoke extract at high concentrations inhibits osteoblast-like cell proliferation and differentiation. On the other hand, smoking carcinogen exposure may increase activity of existing osteoclasts. Both of these would contribute to increased bone resorption^34,35^. Kubota et al.^36^ found augmented alveolar bone loss, delayed alveolar bone recovery and increased number of osteoclasts in a mouse periodontitis model. Smoking changes the life span and apoptosis of periodontal ligament fibroblasts and inhibits the growth and adhesion of fibroblasts, making it difficult to restore damaged periodontal tissues. Smoking can influence the oral environment by inducing hypoxia, lowering the periodontal redox potential and affecting the subgingival micro-environment, which is beneficial to the survival of anaerobic bacteria^37^. Smoking may alter the transcription and methylation status of extracellular matrix-related genes, which may exacerbate periodontitis^38^.

##### The digestive tract

Cigarette smoke and its active compounds impair the fundamental structure of the gastrointestinal tract through the induction of cellular apoptosis and the inhibition of mucosal cell renewal^39^. A number of studies have shown that cigarette smoke can induce cell apoptosis in the esophagus and gastric mucosa, as well as in the inner layers of the small intestine and colon, impairing integrity of the mucous. Smoking can be associated with increased risk for gastrointestinal metaplasia, chronic gastric mucous atrophy, and gastric cancer-like dysplasia^40-42^. Cigarette smoke also interferes with the protective function of the gastrointestinal tract by the contraction and spasm of the gastric submucosal blood vessels, leading to mucosal ischemia and hypoxia. A decrease in gastric mucosal blood flow is an important factor in destruction of the integrity of the gastric mucosa^43^. Smoking can cause disorders of the pyloric sphincter function and hinder the reflux of intestinal fluid. Smoking can also affect prostaglandin synthesis in the duodenal mucosa of the stomach, reducing mucus volume and mucosal blood flow, thereby impairing the defence function of the mucosa and making the gastric mucosa more susceptible to infection. Smoking may affect the closing function of the pyloric sphincter, which can lead to bile reflux and damage to the gastric mucosal barrier. Accumulated bile will stimulate the gastric mucosa to increase gastric acid secretion, causing contraction of blood vessels in the stomach wall and increasing the chance of infection with *H. pylori* (HP).

#### Immunity

The impact of smoking on immunity is complex, with both pro-inflammatory and immunosuppressive effects^44^. Long-term and high-dose exposure of cigarette smoking can significantly damage the immune system and cause an imbalance in the inflammatory response^45^. Under the influence of cigarette smoke, both innate immunity and adaptive immunity can be observed with cell dysfunction and reduced effector molecules^46,47^, eventually leading to the colonization of the pathogen and the occurrence of infections.

##### Smoking and innate immunity

The most important part of innate immunity are innate immune cells, including macrophages, epithelial cells, dendritic cells and natural killer cells etc. A shorttime tobacco smoke exposure can generally stimulate immunity, activating and increasing macrophages and neutrophils with increased inflammatory mediators [tumor necrosis factor (TNF-α), interleukin (IL)-1β, IL-8, and leukotriene B]^45^. Long-term chronic inflammation and cigarette smoke exposure, can damage innate immune cells and inhibit the production of immune molecules, leading to the rapid spread and long-term colonization of pathogens^45,46^. Cigarette smoking suppresses the phagocytosis of bacteria and apoptotic cells in macrophages^48-51^. Cigarette smoking is also associated with a decreased number, inhibited maturation, and reduced expression, of IFN-α in dendritic cells^52-54^. Moreover, cigarette smoking can reduce cytotoxicity and cytoactivity of natural killer cells, decreasing the expression of IFN-γ and TNF-α in natural killer cells^55,56^. Corriden et al.^57^ revealed that e-cigarette inhalation can reduce human neutrophil chemotaxis, phagocytosis, and neutrophil extracellular trap formation. All of these cells and molecules are important in the host defence against infection, thereby smoking increases the risk, severity and duration of infection.

Smoking can also lower the level of some antibacterial molecules. Moore et al.^58^ suggested that cigarette exposure impairs a multifunctional innate defence protein named ‘Short palate lung and nasal epithelial clone 1 (SPLUNC1)’, which is secreted into airway surface liquid by the underlying epithelia and also is a key component of the innate immune response to infections, especially gram-negative organisms. Duffney et al.^59^ found that cigarette smoke exposure impairs antiviral responses in lung epithelial cells by decreasing the phosphorylation of the key antiviral transcription factor, interferon response factor 3. In addition, cigarette smoke can suppress immune activation and the expression and secretion of effector cytokines in peripheral blood mononuclear cells, resulting in a reduced immune response^60^.

##### Smoking and adaptive immunity

Smoking damages adaptive immune cells and reduces the release of effector molecules. Warny et al.^61^ found that smoking status is a risk factor for lymphopenia. Bhat et al.^62^ proved that chronic secondhand smoking impairs B- and T-cell responses and reduces the production of antibodies in both serum and bronchoalveolar lavage samples. Patin et al.^63^ observed that active smoking significantly increased the number of circulating immune cells: circulating CD45+ cells by 23% (99% CI: 11–37%), and conventional lymphocytes by 26% (99% CI: 10– 45%). Cigarettes can reduce human immunoglobulin levels in serum and saliva. Gonzalez et al.^64^ and Nesrin et al.^65^ found decreased IgG levels in smokers. Maria et al.^66^ reported lower levels of salivary IgA, IgG, and IgM in smokers than in non-smokers. Serum IgG and IgM levels will rise significantly after smoking cessation^67^. Cigarette smoke inhibits expression and secretion of effector cytokines, such as IFN γ, TNF and IL-2 proteins, resulting in a reduced immune response60,68.

### Smoking and respiratory infections

Many large national and international studies state that there is sufficient evidence that smoking is closely related to various respiratory infections, with a clear dose-response, including acute respiratory tract infection (ARTI) and particularly communityacquired pneumonia (CAP). The pathogens involved in these findings include bacteria, fungi, viruses, and *Mycobacterium* tuberculosis. Smoking cessation can reduce the risk of morbidity.

#### Smoking and CAP

CAP is a common respiratory infection, and pathogens related to CAP include *Streptococcus pneumoniae, Mycoplasma pneumoniae, Chlamydia pneumoniae,* and several respiratory viruses, among others. Smoking can substantially increase the morbidity and mortality risk of CAP whereas smoking cessation can reduce the risk of morbidity. Baik et al.^69^ reported that the risk of CAP in smokers is approximately 1.5 times that of non-smokers (95% CI: 1. 00–2.14), regardless of sex. For male smokers, who smoke more than 25 cigarettes per day, the rate of CAP can increase 2.54 times (95% CI: 1.40–4.59) that of non-smokers. Almirall et al.^70^ proved that for those who smoked more than 20 cigarettes daily, the risk was increased by 3.89 (95% CI: 1.75–8.64)^70^. As for older people, the risk of CAP in smokers is 2.3–3.1 times that of non-smokers^71,72^. Nuorti et al.^73^ confirmed that smoking is the strongest independent risk factor for invasive pneumococcal disease, and the morbidity of CAP in smokers is 4.1 times that of non-smokers (95% CI: 2.4–7.3). In addition, the CAP risk of passive smokers is 2.5 times that of non-smokers (95% CI: 1.2–5.1). Smoking cessation can reduce the risk of CAP. Compared with individuals who quit smoking for less than 1 year, the risk of CAP has been found to be significantly lower among individuals who had quit smoking for more than 4 years (OR=0.39; 95% CI: 0.17–0.89)^74^. After 5 years of smoking cessation, the risk of CAP in former smokers is nearly the same as that of non-smokers^75^.

#### Smoking and acute respiratory tract infection (ARTI)

Generally speaking, ARTI is caused by viruses (70– 80%), including rhinovirus, coronavirus, adenovirus, influenza and parainfluenza virus, and respiratory syncytial virus. Another 20–30% of ARTIs can be attributed to bacteria. There is sufficient evidence that smoking increases the risk and recurrence rate of upper respiratory tract infections, whereas smoking cessation can reduce these. Cohen et al.^76^ showed that the risk of developing a clinical cold in smokers was 2.08 times that of non-smokers (95% CI: 1.18–3.70). Blake et al.^77^ found that the risk of ARTI among nonsmokers (95% CI: 1.1–1.8) is 1.46 times lower than that of smokers. An et al.^78^ discovered that in those who smoked on 1–4, 5–10, 11–20, or 21–30 days during the previous 30 days, the prevalence of one or more days of cough or sore throat increased by 5.8%, 9.5%, 8.9%, and 11.0%, respectively. Aronson et al.^79^ revealed that smokers are more prone to ARTI, with a longer cough duration (8.9 vs 6.8 days) and an increased risk of lower respiratory illnesses (57% vs 45%). Zhou et al.^80^ reported that passive smokers are 1.59 times more likely to have a cold than non-smokers (95% CI: 1.27–1.99). Guan et al.^81^ revealed that smoking habits among family members is an independent risk factor for recurrent ARTI in children. Smoking in the mother can cause fetal respiratory movement fatigue in the uterus, impair fetal respiratory function, and reduced respiratory defence function.

Smoking can weaken the potency of influenza vaccines, reducing their protective effect and thereby increasing the morbidity of influenza and hospitalization. Godoy et al.^82^ discovered that hospitalization among smokers was 1.32 times that of non-smokers (95% CI: 1.04–1.68). Influenza virus subunit vaccines can help to protect smokers from hospitalization at a rate of 21% (95% CI: -2, 39%) whereas non-smokers have a protection rate of 39% (95% CI: 22–52%). Park revealed that there is a low rate of vaccination among ex-smokers (OR=0.734; 95% CI: 0.63–0.854). In addition, smokers have an increased influenza viral load after infection, which aggravates tissue lesions83. Lee et al.^84^ found that mice exposed to cigarette smoke had faster virus proliferation and higher viral load.

#### Smoking and COVID-19

The current COVD-19 pandemic, which is caused by severe acute respiratory syndrome coronavirus 2 (SARS-CoV-2), has caused more than 370000 deaths^85^. The relationship between smoking and COVID-19 is controversial^86^. Many studies show that smoking increases the incidence and severity of COVID-19. Smoking upregulates angiotensinconverting enzyme 2 (ACE2), the receptor of SARSCoV-2 and SASR-CoV, increasing susceptibility to COVID-19 and Severe Acute Respiratory Syndrome (SARS) infection^87-91^. High expression of ACE2 is also related with cytokine storm and immune system dysfunction, leading to lung injury in both COVID-19 and SARS patients^92,93^. A community-based cohort study of 387109 adults in the UK illustrated that smoking increases the risk of COVID-19 (RR=1.42; 95% CI: 1.12–1.79)^94^. Vardavas and Nikitara^95^ found that smokers were more likely to have more severe symptoms of COVID-19 (RR=1.4; 95% CI: 0.98–2.00) and were more likely to be admitted to the intensive care unit, require mechanical ventilation or die, in comparison with non-smokers (RR=2.4; 95% CI: 1.43–4.04). Liu et al.^96^ concluded that a history of smoking can lead to the progression of COVID-19 pneumonia (OR=14.285; 95% CI: 1.577– 25.000). Guan et al.^97^ analyzed the smoking status in 1099 COVID-19 patients, finding that smoking rate in patients with poor outcomes is higher than that without poor outcomes (25.8% vs 11.8%). In addition, males, with high rates of smoking, tend to have more serious complications in SARS and Middle East Respiratory Syndrome (MERS)^98-101^. This sex predisposition can be observed in COVID-19, which indirectly suggests the relationship between smoking and COVID-19^102^.

However, there are different views. The prevalence of smoking among people with COVID-19 was about 5.4% (95% CI: 3.5–7.7%)^103^, and such a low prevalence may not support that smoking increases the risk of SARS-CoV-2 infection. Simon et al.^104^ found that active smoking was associated with lower odds of having a positive test result, which may be explained by selection bias and affected nasopharyngeal viral load by smoking. A meta-analysis by Lippi^105^ showed no significant association between active smoking and severity of COVID-19 (OR=1.69; 95% CI: 0.41–6.92; p=0.254). But Guo^106^ corrected the meta-analysis of Lippi and found that the pooled OR was 2.20 (95% CI: 1.31–3.67; p=0.003) and the latest meta-analysis by Roengrudee^107^ shows that smoking is a risk of disease progression in COVID-19 (OR=1.91; 95% CI 1.42– 2.59). In conclusion, currently limited epidemiological evidence are insufficient to draw a firm conclusion, but a warning for smoking cessation in the pandemic of COVID-19 is necessary^102,108,109^.

#### Smoking and infection with different respiratory pathogens

Smoking increases the risk of influenza infection and hospitalization. Kark et al.^110^ found that smokers are 1.44 times more likely to have influenza infection than non-smokers (95% CI: 1.03–2.01; incidence rate 60.0% vs 41.6%). Kark et al.^111^ also demonstrated that smokers are 2.49 times more likely to contract influenza than non-smokers (95% CI: 1.56–3.96; incidence rates 68.5% vs 47.2%). Mustafa et al.^112^ revealed that the risk of death for active smokers with influenza is 7.08 times greater than that of other groups. A multicenter case-control study of 2554 people aged >65 years in Spain showed that smoking is associated with an increased risk of hospitalization for influenza (OR=1.32; 95% CI: 1.04–1.68). The effectiveness of influenza vaccination for non-smokers is higher than that of smokers (39% vs 21%)^82^. Heavy smoking history (≥ 20 pack-years) is an independent risk factor for influenza occurrence in hospitalized patients with chronic obstructive pulmonary disease (COPD) (OR=3.056; 95% CI: 1.072–8.715; p=0.037)^113^.

Smoking can increase the morbidity of pneumococcal disease. Research by Flory et al.^114^ showed that the incidence of streptococcus pneumonia in non-smokers is 5.1/100000 (95% CI: 4.2–6.2), and the incidence in smokers is significantly higher at 16.3/100000 (95% CI: 14.6–18.1), the risk ratio of smoking is 2.2 (95% CI: 1.7–3.0), and the attributable risk is 35.6%. A study in Australia also found that smokers are 3.7 times more likely to develop Streptococcus pneumonia than non-smokers^115^.

Smokers are more likely to develop *Legionella* pneumonia than non-smokers. Straus et al.^116^ confirmed that the risk of *Legionella* pneumonia among smokers is 3.75 times that of non-smokers (95% CI: 2.27–6.17). The greater the daily smoking amount the higher the risk of *Legionella* pneumonia. Research by Doebbeling et al.^117^ also showed that smoking is an independent risk factor of *Legionella* pneumonia.

The risk of pulmonary fungal disease, such as coccidioidomycosis, cryptococcosis etc., is considerably increased in smokers. In Italy, a study conducted in 33 medical centers revealed that smoking can increase the risk of cryptococcosis among people with and without HIV^118^. Rosenstein et al.^119^ found that smoking in the previous 6 months is an independent risk factor of severe coccidioidomycosis, with a ratio of 2.4 (95% CI: 1.1–5.4).

Smoking exposure during pregnancy may cause disease by altering RSV and changing neonatal immune responses, which can increase the risk of respiratory tract infection. Cheemarla et al.^120^ showed that intrauterine smoke exposure in a mouse model led to increased lung inflammation, with increased neutrophil influx into the airway of infected mice and delayed virus clearing; however, the authors found that RSV-specific CD8^+^ T cells had a decreased response.

#### Smoking and tuberculosis infection

Both active and passive smoking can increase the risk of tuberculosis. The World Health Organization 2007 Global Tuberculosis Report clearly pointed out that smoking is one of the five major risk factors for tuberculosis infection. In 830000 patients newly diagnosed with tuberculosis, the increased risk of contracting the disease is attributed to smoking. The morbidity of tuberculosis in smokers is 1.6–2.5 times that of non-smokers^121^; a similar conclusion has been reached in several large meta-analyses^122-124^. Lin et al.^125^ demonstrated that smokers were 1.94 times more likely to have active tuberculosis than nonsmokers (95% CI: 1.01–3.73). Oztürk et al.^126^ proved that tobacco abuse (over 20 packs/year) and smoking from a young age (<16 years) is the key factor of active tuberculosis. David et al.^127^ found that the incidence of latent tuberculosis infection (LTBI) in non-smokers, previous smokers, and current smokers is 4.1%, 6.2%, and 6.6%, respectively; the risk of LTBI among non-smokers (95% CI: 1.1–2.9) was 1.8 times lower than that of current smokers. Bronner et al.^128^ conducted a case-control study in 146 patients from South Africa with tuberculosis and HIV coinfection. The authors discovered that the median CD4 cell count of the smoking group was lower (60/mm^3^ vs 81/mm^3^) and the median viral load higher (173/μL vs 67/μL) among current and past smokers with HIV, and the risk of tuberculosis in these individuals was 3.2 times (95% CI: 1.3–7.9) and 1.8 times (95% CI: 0.8–4.4) that of non-smokers with HIV.

There is evidence that smoking can adversely affect the therapeutic efficacy and prognosis of tuberculosis. Leung et al.^129^ found that current smokers with a history of tuberculosis have a significantly higher risk of tuberculosis recurrence than non-smokers (OR=2.48; 95% CI: 1.04–5.89). Abal et al.^130^ revealed that the duration of sputum negative conversion among smokers with tuberculosis is significantly prolonged. Tan et al.^131^ revealed that, with the same treatment, the sputum negative conversion rate among smokers is significantly lower than that of non-smokers. Chuang et al.^132^ confirmed that smokers have a higher rate of failure in tuberculosis treatment (33%) and a more serious x-ray classification of lung lesions. Smokers require a longer treatment period to convert the sputum culture from positive to negative (HR=1.12; 95% CI: 1.03–1.39). Xiong et al.^133^ reported that the lesion absorption rate in their non-smoking group (85.7%) was significantly higher than in the smoking group (54.5%). One possible reason for this is that smokers have lower serum albumin levels, which results in a lack of protein to supply lesion repair during treatment and slows sputum negative conversion. A significant increase in the mortality rate among smokers with tuberculosis has been reported. A prospective cohort study of 486341 adults in Taiwan showed that the tuberculosis mortality rate among smokers is 9 times that of non-smokers^134^. In India, Gajalakshmi et al.^135^ found that people with a history of smoking are 4.5 times more likely to die from tuberculosis than nonsmokers (RR=4.5; 95% CI: 4.0–5.0; the attribution rate of smoking is 61%).

### Smoking and digestive system infections

#### Smoking and Helicobacter pylori (HP) infections

HP infection is an important risk factor for digestive diseases such as peptic ulcer, chronic gastritis, and gastric cancer. Smoking is an independent risk factor for HP infection, which can increase the morbidity of HP infection and lead to a poor radical treatment. In a study from China, the HP infection rate in the smoking group (51.01%) was significantly higher than that in the non-smoking group (38.27%)^136^. Another study also showed that the HP infection rate among smokers was significantly higher than that of non-smokers, and smoking is an independent risk factor for HP infection^137^. Chen et al.^138^ revealed that smoking can cause failure of anti-HP treatment. Itskoviz et al.^139^ demonstrated that the failure of HP radical treatment is positively correlated with current smoking (OR=1.15; 95% CI: 1.10–1.20).

#### Smoking and Clostridium difficile infections (CDI)

Clinically, CDI can cause mild diarrhea to bloody diarrhea, which can lead to pseudomembranous colitis, toxic megacolon, intestinal necrosis, and can even be life-threatening. Smoking increases the risk of CDI and is associated with poor prognosis. Rogers et al.^140^ discovered that the risk of CDI among former smokers was increased by 33% in comparison with non-smokers, whereas in current smokers, the risk of CDI was increased by 80%. Barker et al.^141^ found that infection in patients with a history of smoking was significantly less likely to be cured within 2 weeks than in non-smokers. Ruiter et al.^142^ conducted a retrospective cohort study in a national sample of pregnant women and showed that smoking increased the diagnostic rate of CDI in pregnant women who were hospitalized (adjusted OR, AOR=1.4; 95% CI: 1.2–1.7). Garg et al.^143^ revealed that smoking increases the chance of emergency CDI hospitalization.

#### Smoking and anal abscess

Studies have shown that smoking increases the risk of anal abscesses. Zheng et al.^144^ reported that smoking is associated with anal abscess and anal fistula disease (OR=12.331; 95% CI: 8.364–18.179). Devaraj et al.^145^ proved that the risk of anal abscess/anal fistula among military veterans with a smoking history within 1, 5 and 10 years was 2.15, 1.72, and 1.34 times that of non-smoking veterans, respectively. These findings suggest that a recent smoking history (within 1 year) is a significant risk factor for anal abscess/anal fistula (OR=2.15; 95% CI: 1.34–3.48). Wang et al.^146^ also discovered that smoking is an independent risk factor for anal fistula (OR=4.300; 95% CI: 3.640–5.080).

### Smoking and genital tract infection

#### Smoking and human papillomavirus (HPV) infection

The main types of HPV are HPV 1, 2, 6, 11, 16, 18, 31, 33 and 35; cervical cancer may be related to long-term infection of HPV16 and 18. Smoking can increase the morbidity of HPV infection in both man and women. A multicenter randomized controlled trial^147^ conducted among women in the United States showed that smoking increases the risk of HPV infection by lowering the immune response. Current smokers have a higher risk of infection with HPV16 (OR=1.29; 95% CI: 1.11–1.73) than non-smokers. Women who smoke more than 10 cigarettes per day (OR=1.59; 95% CI: 1.27–2.15) are at higher risk than those who smoke less than 10 per day (OR=1.09; 95% CI: 0.94–1.44). Feng et al.^148^ found that passive smoking does not cause a continually increased risk of HPV infection (OR=1.11; 95% CI: 1.00–1.24). In men, Liu et al.^149^ demonstrated that smoking can result in HPV infection (adjusted prevalence ratio, APR=3.58; 95% CI: 1.81–7.08) and can lead to high-risk HPV infection (APR=4.08; 95% CI: 1.52–10.94). Another study on the prevalence of oral HPV in patients with special healthcare needs found that both current and former smokers have a higher risk of developing oral HPV infection, and smokers have higher HPV viral loads than non-smokers^150^.

#### Smoking and Trichomonas vaginalis (TV) infection

Infection with TV is the most common curable sexually transmitted infection (STI) in the world and can increase the risk of pelvic inflammatory disease (PID), HIV transmission and premature delivery. In Brazil, a survey on the risk factors for TV infection showed that smoking increases the risk of infection with TV (AOR=1.66; 95% CI: 1.04–2.64)^151^. In Kenya, a 2-year longitudinal study of STIs among 350 female sex workers found that smoking history is a risk factor for increased incidence of TV within 2 years (HR=2.66; 95% CI: 1.24–5.73)^152^.

#### Smoking and mycoplasma and chlamydia infection in the genital tract

*Mycoplasma genitalium* and *Chlamydia trachomatis* (CT) infections are associated with female genital diseases, including cervicitis, urethritis, PID, infertility, ectopic pregnancy, adverse delivery outcomes, and HIV infection. Balkus et al.^153^ proved that smoking is independently associated with an increased risk of genital mycoplasma infection (AHR=3.02; 95% CI: 1.32–6.93). Adamma et al.^154^ conducted a cohort study among 954 young women; the results showed that smoking increases the incidence of genital chlamydia infection (RR=2.2; 95% CI: 1.2–3.9).

### Smoking and other infections

#### Skin infections

A retrospective cohort study showed that smoking during pregnancy can increase the incidence of skin infections in those aged <17 years (HR=1.21; 95% CI: 1.13–1.30), especially for children under 1 year of age. Smoke-free pregnancy can reduce the rate of hospitalization for skin infections in children by 9.6%. Smoking during pregnancy may lower the child’s immunity and increase the risk of infection155.

#### Otitis media

Both active and passive smoking can cause acute otitis media, recurrent otitis media, and middle ear effusion in children. In 2006, the U.S. Surgeon General’s Report pointed out that children whose parents smoked were 1.0–1.5 times more likely to have acute otitis media than those whose parents did not smoke. The rate of recurrent otitis media in children whose parents smoked was 1.37 times higher than that of children whose parents did not smoke (95% CI: 1.10–1.70). In 2013, a cohort follow-up study in 400 mother-child pairs showed that smoking habits of mother (current habit and former habits; HR=2.00; 95% CI: 1.19–3.36) can cause otitis media in children aged <4 years (HR=1.17; 95% CI: 1.05–1.31)^156^. Studies have shown that exposure to cigarette smoke among exposure of pregnant women is associated with an increased risk of otitis media in infants. A study of 72 patients with an acute ear infection showed that the risk of acute ear infection in infants is closely related to the daily smoking amount of mother during pregnancy, as follows: 1–9 cigarettes (OR=1.6; 95% CI: 1.1–2.5), 10–19 cigarettes (OR=2.6; 95% CI: 1.6–4.2), more than 20 cigarettes (OR=3.3; 95% CI: 1.9–5.9). The risk of subacute ear infection in infants is also closely related to the mother’s daily smoking amount: 10–19 cigarettes (OR=2.6; 95% CI: 1.4–5.0), and >20 cigarettes (OR=2.8; 95% CI: 1.3–6.0)^157^.

#### Periodontal infections

Smoking can cause an increased rate of periodontal infection. Ojima et al.^158^ studied the data from two national adult sample surveys (dental disease survey and national nutrition survey) in Japan. They found that the prevalence of periodontitis and other severe periodontal infection in non-smokers, former smokers, and current smokers was 39.3% and 49.5%, 47.3% and 7.9%, 11.7% and 12.4%, respectively. Among people aged >40 years, the risk in current smokers of general periodontitis and more severe periodontal infection was 1.38 times (95% CI: 1.12–1.71) and 1.40 times (95% CI: 1.04–1.89) that of non-smokers, respectively. Tomar et al.^159^ reported that the rate of periodontitis among current smokers is 3.97 times that of non-smokers (95% CI: 3.20–4.93); as for previous smokers, the rate is 1.68 times that of non-smokers (95% CI: 1.31–2.17). Another study showed that the risk of periodontitis in those who smoke fewer than 9, 10–19, 20, 21–31 and >31 cigarettes per day was 2.79 times (95% CI: 1.90–4.10), 2.96 times (95% CI: 2.12–4.14), 4.72 times (95% CI: 3.46–6.43), 5.10 times (95% CI: 3.48–7.47) and 5.88 times (95% CI: 4.03–8.58) that of non-smokers, respectively. The risk of periodontitis in patients who have quit smoking for <2, 3–5, 6–10, and >11 years is 3.22 times (95% CI: 2.18–4.76), 2.27 times (95% CI: 1.26–4.07), 1.99 times (95% CI: 1.23–3.21) and 1.15 times (95% CI: 0.83–1.60) that of non-smokers, respectively.

Smoking can lead to severe periodontitis and increased periodontal injury. Zhang et al.^160^ demonstrated that smoking is an independent risk factor for the onset of severe periodontitis in the elderly, and the morbidity of severe periodontitis in smokers is 3.160 times that of non-smokers (95% CI: 1.051–1.310). Kinane et al.^161^ confirmed that smokers have decreased periodontal pocket depth and adhesion levels than non-smokers after non-surgical or surgical treatment. Zhao et al.^162^ proved that the proportion of periodontitis is increased in smokers, and the greater the degree of smoking, the deeper the periodontal pockets and the greater the attachment loss.

#### Bacteremia

Studies have shown that the incidence of bacteremia in smokers is significantly increased, which is an important risk factor for poor prognosis and mortality of bacteremia^163^. In a retrospective analysis of the clinical data of 67 patients in a Spanish hospital with positive blood cultures for *Streptococcus pneumoniae* from January 1999 to October 2010, investigators found that smoking was the main risk factor for pneumococcal bacteremia^164^.

#### HIV Infection

Studies have shown that smokers have a higher rate of HIV infection and morbidity of AIDS. Mdodo et al.^165^ reported that in patients with HIV, 42.4% (95% CI: 39.7–45.1%) were current cigarette smokers, 20.3% (95% CI: 18.6–22.1%) were former smokers, and 37.3% (95% CI: 34.9–39.6%) had never smoked. A 7.9-year longitudinal study in 924 women with low socioeconomic status found that smokers have a higher risk of developing AIDS (HR=1.36; 95% CI: 1.07–1.72) and a higher mortality rate. Smokers display weak viral immune responses and are more likely to have failure of immunotherapy^166^.

There are also increased opportunistic infections and a decreased treatment efficacy among HIVinfected smokers^167^. Clinical consequences of the cumulative effects of HIV infection and smoking may increase the risk of pneumonia, COPD, cardiovascular disease and non-AIDS cancer among smokers^168-174^. Cigarette smoking may have a cumulative immunosuppressive effect in HIV patients. Compared with HIV-infected non-smokers, a marked depression in lung CD4^+^ and CD8^+^ lymphocytes and suppressed spontaneous production of the proinflammatory cytokines IL-1β and TNF-α can be observed in HIVinfected smokers^167,175^. Smoking in HIV infected people can lead to impaired function of CD4^+^ and CD8^+^ T cells and increased intestinal bacterial translocation^176^. These may explain why smoking can lead to increased opportunistic infections like oral candidiasis, esophageal candidiasis and pneumocystis pneumonia in HIV-positive patients^177^. In addition, cigarette smoking is associated with decreased efficacy of treatment, resulting in reduced immunological reaction^167^.

### Smoking and surgical site infection (SSI)

Smoking increases the incidence of SSI. Forsythe et al.^178^ stated that smoking status (OR=1.7; 95% CI: 1.4–1.9) is an independent risk factor for SSI development. Baier et al.^179^ also revealed that smoking was an independent risk factor for postoperative SSI (OR=2.22; 95% CI: 1.27–3.90). One study showed that after surgery for adult spinal deformity and distal femoral fractures, the wound infection risk in the smoking group was higher than that of the non-smoking group^180,181^. For patients with diabetes, smoking is a risk factor of postoperative infection. Peng et al.^182^ proved that smoking is a risk factor for wound infection after lumbar surgery in patients with diabetes (OR=3.830; 95% CI: 1.003–6.684); however, 4 weeks of strict smoking cessation prior to surgery reduced the risk of wound infection. Smoking results in an increased risk of infection at other sites after surgery with a prolonged hospitalization time and increased expenses. In patients after total hip arthroplasty (THA), Debbi et al.^183^ found that the hospitalization time and the total hospitalization costs were significantly increased in smokers. Matharu et al.^184^ discovered that compared with non-smokers and former smokers, current smokers have an increased risk of lower respiratory tract infection after primary THA and total knee arthroplasty. Additionally, a retrospective study of 206 transsexual men who underwent testicular implantation between January 1992 and December 2018 found that smoking history was a risk factor for postoperative infection and prosthetic transplantation^185^.

## CONCLUSION

Smoking can substantially increase the incidence and the mortality of infections in a clear dosedependent manner. In addition, smoking leads to a poor prognosis. The recurrence rate and uncured rate of infection in smokers are higher than in nonsmokers. Smoking cessation can reduce damage owing to smoking and reduce the risk of infection. Active smoking cessation promotion and education are practical and cost-effective measures to prevent the risk of infection caused by smoking and to reduce the incidence rate and mortality of infection. Encouraging smokers to be vaccinated is also important to avoid infection.
